# Advantages of the Surface Structuration of KBr Materials for Spectrometry and Sensors

**DOI:** 10.3390/s18093013

**Published:** 2018-09-09

**Authors:** Natalia Vladimirovna Kamanina, Svetlana Vladimirovna Likhomanova, Pavel Viktorovich Kuzhakov

**Affiliations:** 1 Laboratory for Photophysics of Media with Nano-Objects, Vavilov State Optical Institute, Kadetskaya Liniya V.O., dom 5/2, St.-Petersburg 199053, Russia; lsv-87@bk.ru (S.V.L.); kpv_2002@mail.ru (P.V.K.); 2 Electronics Department, St.-Petersburg Electrotechnical University (“LETI”), Ul.Prof.Popova, dom 5, St.-Petersburg 197376, Russia

**Keywords:** potassium bromide, carbon nanotubes, laser-oriented deposition method, surface structuration, spectra, mechanical and wetting properties

## Abstract

A potassium bromide (KBr) material, which has been widely used as the key element in Fourier spectrometers and as the output window of the IR-lasers, was studied via applying carbon nanotubes in order to modify the potassium bromide surface. The laser-oriented deposition method was used to place the carbon nanotubes at the matrix material surface in the vertical position at different electric fields varying from 100 to 600 V × cm^−1^. The main idea of the improvement of the spectral properties of the potassium bromide structure is connected with the fact that the refractive index of the carbon nanotubes is substantially less than the refractive index of the studied material, and the small diameter of the carbon nanotubes allows one to embed these nano-objects in the voids of the lattice of the model matrix systems. Moreover, the mechanical characteristics and wetting features of potassium bromide structures have been investigated under the condition mentioned above. Analytical and quantum-chemical simulations have supported the experimental results.

## 1. Introduction

Presently, one of the main basic approaches to improve the properties of large groups of optical inorganic materials is to use carbon nanotubes (CNTs) in order to modify the materials’ surface. This is due to the fact that the refractive index of the CNTs [1,2,3] is substantially less than that of most inorganic materials, and the diameter of CNTs allows one to embed nano-objects in the voids of the lattice of matrix systems. This homogenizes the interfaces with a very small (air) and relatively large (matrix materials, in which surfaces the CNTs are incorporated) refractive index, which causes a change in the range of transmission, at least. Moreover, these nano-objects possess the strong hardness of their C–C bonds [4,5]. The Young’s modulus of CNTs is close to some units of TettaPascals, which can increase the mechanical features of optical inorganic materials as well.

Considering this, potassium bromide (KBr) materials can be chosen as a good model matrix. These materials can be advantageously applied as different optoelectronic elements, including as the output window of an IR-laser, as electro-optical fibers, sensors, etc. KBr is transparent in the spectral range of 0.25–25 microns [6], which prompts its use not only in optoelectronics but also in the biomedicine applications. The refractive index of KBr materials changes from 1594 (at the wavelength of ~0.391 microns) to 1461 (at the wavelength of ~25,140 microns). Unfortunately, the hydrophilicity of this material often creates problems. This material is soluble in water, acetone, methanol, ethanol, glycerin, and other solvents. Thus, the surface of KBr crystals should be protected from drops of water. This problem can be solved by using the nanostructuration process, by which CNTs can be placed on the KBr surface in the vertical position by application of the laser-oriented deposition (LOD) technique [7]. 

In the current paper, the spectral, mechanical, and wetting properties of potassium bromide materials were studied by applying carbon nanotubes as effective nano-objects and using the laser-oriented deposition technique as the prospective technical method.

## 2. Materials and Methods

To modify the properties of KBr materials via their surface treatment, single-wall carbon nanotubes (SWCNTs), type #704121, with a diameter in the range of 0.7–1.1 nm, purchased from Aldrich Co. (Karlsruhe, Germany), were used. The dimensions of the nanotubes were important in order to combine the CNTs’ diameter directly with the elementary lattice of the model material. Moreover, Russian CNTs and nanofibers, type “Taunit-MD”, from the Tambov Company “Nanotech-Center” (Tambov, Russia) production were applied. 

**Figure 1:**
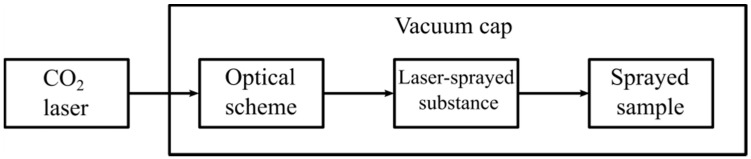
Principle scheme of the laser-deposition technique.

To modify the potassium bromide surface, an IR CO_2_-laser with *p*-polarized irradiation at the wavelength of 10.6 μm and with the power of 30 W was used. The general view of the block scheme is shown in Figure 1. One can see that the laser system is connected with a vacuum hood, which contains the fixing unit samples and the device for placing the substances deposited on the substrate.

Moreover, the CNTs were placed at the materials’ interface under conditions in which an additional electric field of 100–600 V × cm^−1^ was applied in order to orient the nanotubes in the vertical position during the deposition process. Thus, the laser-oriented deposition method [7] was realized efficiently. The above-indicated approach permits varying the velocity of the CNTs and the formation of covalent bonding between the carbon atoms and the surface matrix material atoms. It should be noted that the laser-oriented deposition (LOD) method does not require the creation of any additional conditions for heating of the substrates and the composition of the gas reagents, which favorably differs from the classical methods based on the Chemical Vapor Deposition and Physical Vapor Deposition processes.

The spectra of the nano-object-treated materials were obtained using the Perkin-Elmer Lambda 9 and the Furrier FSM-1202 instruments (Firm “Nica-Garant+”, Saint-Petersburg, Russia) as well as using the VIS SP-26 spectrophotometer (“LOMO” Co., Saint-Petersburg, Russia) operated in the spectral range of 250–1200 nm. A POLAM-P312 microscope (“LOMO” Co., Saint-Petersburg, Russia) was applied to obtain the image of the pure materials and the materials treated with the CNTs. The microhardness was measured with a PMT-3M device, which was produced by “LOMO” Co. (Saint-Petersburg, Russia) with the ability to vary indenter forces as well. Particular emphasis was placed on observing the relief at the material surface via checking the wetting angle. In this case, the OCA 15EC device, purchased from LabTech Co. (Saint-Petersburg-Moscow, Russia), was used to carefully control the wetting angle changes. Additionally, analysis of the modified surface was performed with the Solver Next atomic force microscope AFM (purchased from NT MDT Co., Zelenograd, Moscow region, Russia). It should be mentioned that it permits registering the homogeneity of the surfaces and estimating their roughness under the condition of the nanostructuring process as well. 

## 3. Results and Discussion

It should be noted that the first study of KBr materials successfully treated with CNTs by the LOD technique was presented in [8]. However, the discussion basically focused on the influence of humidity on the transmittance spectra of KBr structures in the IR spectral range, namely, in the wavelength of 4–24 μm. The spectra from UV to VIS were not analyzed and presented, the microhardness parameters and atomic force microscope (AFM) images were not considered, and simulation by the quantum-chemical approach was not performed. Thus, novel results are presented and shown in the current paper. These new data can extend the knowledge about the tendency of the covalent bonding influence between carbon atoms and model material surface atoms on the basic physicochemical parameters of KBr structures.

**Figure 2:**
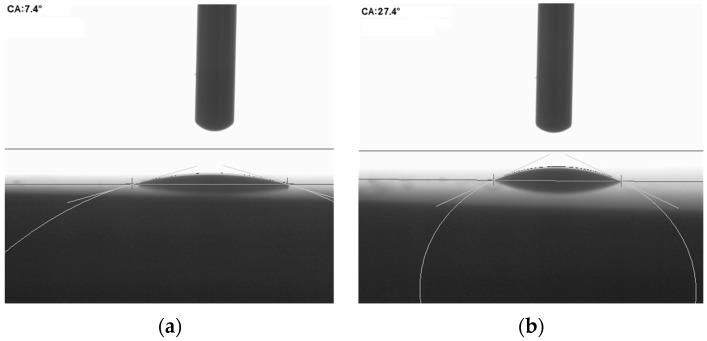
The value of the wetting angle of the KBr crystal before (**a**) and after (**b**) the nanostructuring process.

Some results which show the wetting angle change for the KBr materials via their surface nanostructuration are shown in Figure 2. The presented sample has been chosen from 12 randomly selected samples with the similar parameters. One can see that the wetting angle increased from ~7º up to 27º after applying the CNTs for the laser-oriented deposition technique. This affords protection from drops of water when the KBr material is used in Fourier spectrometer devices or in specific sensitive electro-optical sensors.

**Figure 3:**
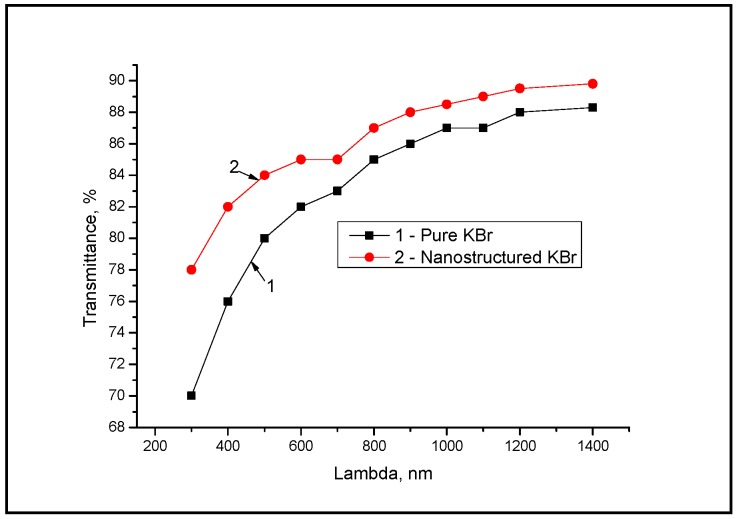
KBr sample transmittance in the UV–VIS spectral range.

Furthermore, the transmittance of KBr materials can be increased after the use of the LOD procedure. The data for the UV–VIS spectral range are shown in Figure 3.

**Figure 4:**
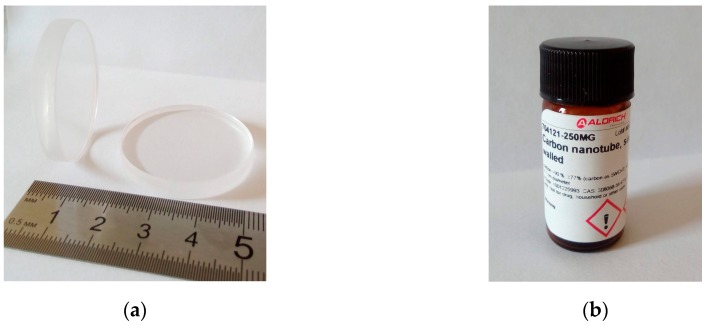
KBr substrates (**a**) and the carbon nanotube (CNT) mixture, type #704121 (**b**).

It should be mentioned that KBr materials have shown an increase in transparence from 70% to 78% at the wavelength of 350–400 nm and from 87% to 89% in the range of 1000–1300 nm. The increase of the tested transparency was from 88.3% to 89.8% in the spectral range of 1400–2400 nm. It should be noted that in order to study the structuration process influence on the physicochemical characteristics of the KBr materials, the substrates with polished surfaces and with a thickness of 5 mm and a diameter of 30–35 mm were used. Figure 4 shows the KBr substrate and the CNT mixture in the vial.

In order to support the transmittance spectra increase, the analytical consideration based on the Fresnel loss decrease can be made. For example, since there is good evidence that the Fresnel losses can be changed under the nanostructuration process, this can be easily shown based on the model glass substrate. It should be mentioned that the refractive indices of ordinary glass and KBr materials are close to each other if one can eliminate the dependence of the refractive index on the wave length. Using the classical method to reduce losses during the reflection process and, thus, to improve the light transmission (aperture ratio) of the optics, the surface of the glass can be subjected to special treatment, which is called “enlightenment of optics”. The thin film should be placed on the surface of the matrix materials (glass), the refractive index of which should be less than the refractive index of glass, namely: $n_{\mathrm{film}}=\sqrt{n_{\mathrm{glass}}}$. In order to minimize reflection losses, the film must have a certain thickness, which can be calculated by the formula: $h=\frac{d}{n_{\mathrm{film}}}$, where *h*—the geometric thickness of the film, *d*—the optical thickness of the film obtained as $d=\frac{\lambda }{4}$, and *λ*—the wavelength of the light in that part of the spectrum, where it is necessary to obtain the maximum of the transmittance. It is well known that a film with a thickness of $\frac{\lambda }{4}$ from a substance with a refractive index of $\sqrt{n_{\mathrm{glass}}}$ decreases the reflective coefficient dramatically. If one considers the interface, such as air–glass, with the reflective index of the glass material to be close to 1.5, the reflection from one surface of the substrate is approximately 4%, and from two surfaces, it is approximately 8%. 

According the interface of CNT/KBr, using the innovative structuration by the LOD approach and forming the covalent bonding between the nano-objects (carbon nanotubes) with the little refractive index *n* of ~1.1 and the larger refractive index of the studied matrix substrate (*n* of the KBr material can be placed in the range of 1.46–1.59 and is dependent on the wavelength), the Fresnel losses via the reflection can be changed by one order of magnitude, at least, which can effectively provoke the change of transmittance. 

The correlation between spectral change and mechanical parameters improvement was found as well. One can see that the microhardness of the KBr structure can be increased substantially via the placement of the CNT at the vertical position on the KBr surface under the LOD procedure. The corresponding results are shown in Table 1. One can see that the microhardness increased up to ~6% after applying the LOD technique. This result was obtained when only one KBr surface was treated. However, this value can be increased even more if not one but two surfaces of the KBr model material have been nanostructured.

**Figure 5:**
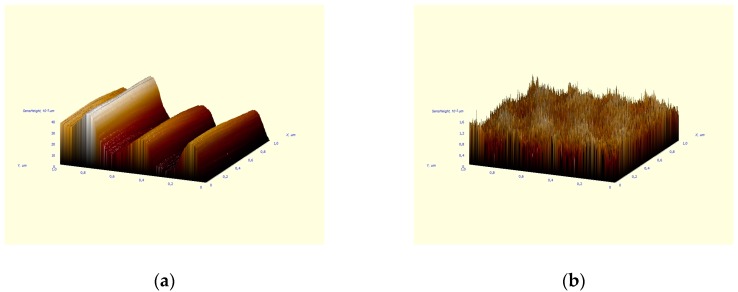
KBr substrates: pure (**a**) and covered with the CNTs (**b**).

It should be mentioned that mechanical properties improvement can efficiently coordinate with the AFM images. Figure 5 indicates the surface of the pure and treated KBr surface by CNTs.

It can be seen that the application of CNTs in the use of LOD technology allows one to grind the surface heterogeneity of the matrix material, reducing the surface defects.

**Figure 6:**
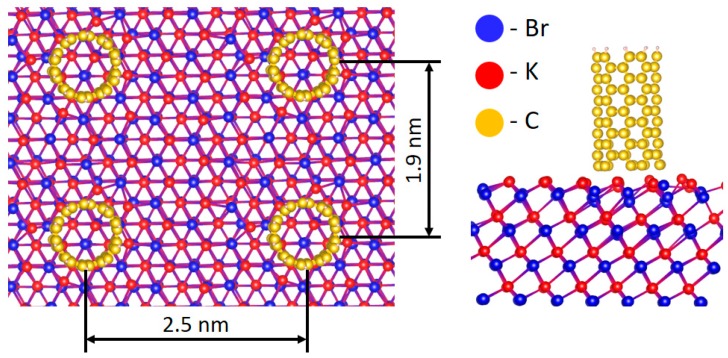
Atomic structure of the considered KBr/CNTs interfaces.

In order to support the experimentally obtained results and to visualize the influence of the CNTs on the KBr surfaces, a quantum-chemical calculation was made using the approach proposed in the papers [9,10,11], which was based on the density functional theory, implemented in the Vienna Ab initio Simulation Package (VASP) package with the projector augmented wave method. For the considered interfaces, the density of the electronic states was calculated. The interface based on the CNT and KBr substrate (namely CNT/KBr) was carried out. The atomic structure of the considered interfaces is presented in Figure 6. According to the data shown in Figure 6, the CNT has been adsorbed on the (1 1 1) KBr surface with two possible types of the termination: with K (red) and Br (blue) atoms, respectively. It has been found that CNT adsorption leads to changes in the surface of KBr structures caused by the strong interaction between the substrate atoms and the carbon atoms in the CNTs. 

**Figure 7:**
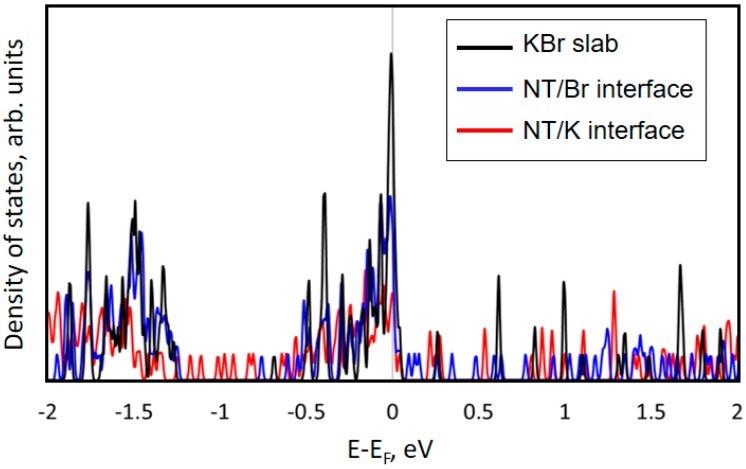
The density of the electronic states for all considered interfaces.

For all considered interfaces, the density of the electronic states was calculated, the results of which are shown in Figure 7. The red line indicates the CNT/K interface, the blue line relates to the CNT/Br interface, and the black line shows the density of the states for the pure nontreated KBr substrate. It was found that the presence of carbon nanotubes on the KBr surface leads to the formation of additional energy levels. For example, in the case of K termination, formation of the additional peaks in the energy range from −1.5 to −0.5 eV took place, while in the absence of carbon nanotubes, there were no peaks found in this energy range. The same calculations were carried out in the case of Br termination (the blue lines).

**Figure 8:**
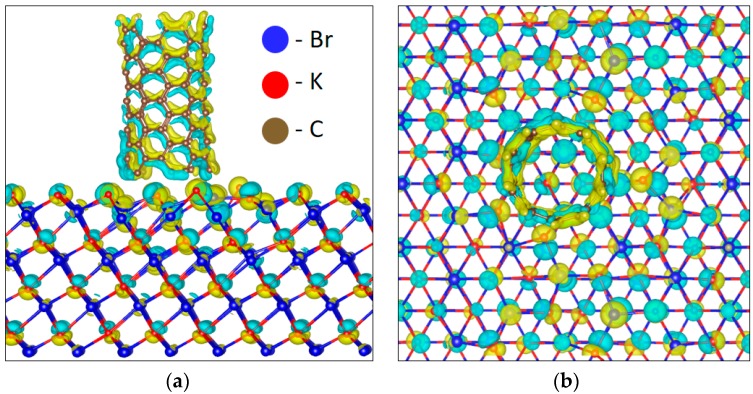
Electron density distribution on the KBr/CNT interface. (**a**) side view of KBr/CNT interface, (**b**) view from above of KBr/CNT interface.

Moreover, the calculation of the electron density distribution for the considered interfaces was carried out, the results of which are presented in Figure 8, which permits to see the distribution of the electron density in the different geometric configurations. The blue and yellow colors depict the increasing and decreasing of the negative charge. The obtained results confirmed the data from the calculations of the density of the states. Based on these data, it can be seen that the redistribution of electron density directly on the KBr/CNT interface changed the electronic properties of the whole structure by means of the formation of additional electronic levels.

Due to the large size of the considered system, the electronic density of the states was calculated only for the small area. Thus, the considered structures consisted of 96 Br atoms, 96 K atoms, and 110 carbon atoms, along with 10 hydrogen atoms in the CNT. 

Thus, the calculation reveals the change of the electronic levels of the KBr materials under the conditions where CNTs were deposited on the matrix surfaces using the laser-oriented deposition technique. The appearance of additional energy levels, therefore, directly shows the change of the refractive parameters. However, the refractive index change naturally provokes the change of the spectral parameters as well. This interrelation is revealed by means of the calculations based on the experimental data. It should be noted once again that, based on these data, one could claim that the redistribution of the electron density directly on the KBr/CNT interface changes the electronic properties of the whole structure by means of the formation of additional electronic levels. It connected with the spectral and mechanical properties improvements obtained by experiments and coincided with the results obtained before for other materials [12,13], for example, based on the ITO (heterostructure of the indium and tin oxides) coatings structured by CNTs [14]. 

Thus, the KBr materials’ properties can be dramatically improved by applying the innovative LOD technique and CNTs as the perspective nano-objects.

## 4. Conclusions

To summarize the obtained experimental and theoretical results, one can conclude the following:

1. The KBr materials can be considered as the perspective model matrix to obtain the correlation between spectral, mechanical, and wetting properties changes by the nanostructuration of their surfaces with the LOD technique.
2. The tendency to form bonds between the carbon atoms and the model materials’ surface atoms has been supported experimentally and theoretically via extending the consideration of the KBr matrix.
3. The transmittance, mechanical, and wetting characteristics of the KBr materials can be increased, which extends the applicable area of such kinds of optical materials in Fourier spectroscopy, in modulation and sensors schemes, in IR-laser use, and in biomedical techniques as well.
4. The data presented can extend the range of the functional materials, the basic properties of which can be successfully varied and optimized namely via structuration of their surfaces.
5. The observed data can be used in the education process because the basic parameter changes can be easily visualized and supported using optoelectronic devices.

## Figures and Tables

**Table 1:** Comparative data of the KBr microhardness change (Indentor was 10 g).

Material Studied	Middle Value of the Microhardness, Pa × 10^9^	Increasing Coefficient of the Microhardness Change, %
Pure KBr	0.00865	~6
KBr structured with CNTs	0.00918	

## References

[1] Fa W., Yang X., Chen J., Dong J. (2004). Optical properties of the semiconductor carbon nanotube intramolecular junctions. Phys. Lett..

[2] Yang Z.P., Ci L., Bur J.A., Lin S.Y., Ajayan P.M. (2008). Experimental observation of an extremely dark material made by a low-density nanotube array. Nano Lett..

[3] Taherpour A.A., Aghagolnezhad-Gerdroudbari A., Rafiei S. (2012). Theoretical and quantitative structural relationship studies of reorganization energies of [SWCNT (5,5)-Armchair-CnH20] (*n* = 20–310) nanostructures by neural network CFFBP method. Int. J. Electrochem. Sci..

[4] Robertson J. (2004). Realistic applications of CNTs. Materialstoday.

[5] Namilae S., Chandra N., Shet C. (2004). Mechanical behavior of functionalized nanotubes. Chem. Phys. Lett..

[6] Rabinovich V.A., Khavin Z.Y. (1978). Brief Chemical Handbook.

[7] Kamanina N.V., Kuzhakov P.V., Vasilyev P.Y. (2013). A Protective Coating for Hygroscopic Optical Materials Based on Laser-deposited Carbon Nanotubes for the Purpose of Optoelectronics and Medical Equipment. Russia Patent.

[8] Kuzhakov P.V., Kamanina N.V. (2014). Spectral Investigations and Wettability of Nanostructured Potassium Bromide, Sodium Chloride, and Magnesium Fluoride Single Crystals. Opt. Spectrosc..

[9] Kresse G., Furthmüller J. (1996). Efficient iterative schemes for ab initio total-energy calculations using a plane-wave basis set. Phys. Rev. B.

[10] Kresse G., Furthmüller J. (1996). Efficiency of ab-initio total energy calculations for metals and semiconductors using a plane-wave basis set. Comput. Mater. Sci..

[11] Blöchl P.E. (1994). Projector augmented-wave method. Phys. Rev. B.

[12] Kamanina N.V. (2014). Features of the Optical Materials Modified with the Effective Nanoobjects: Bulk Properties and Interface.

[13] Kamanina N.V., Bogdanov K.Y., Vasilyev P.Y., Studeonov V.I. (2010). Enhancing the mechanical surface strength of “soft” materials for the UV and IR ranges and increasing their transmission spectrum: Model MgF_2_-nanotube system. J. Opt. Technol..

[14] Kamanina N.V., Zubtcova Y.A., Kukharchik A.A., Lazar C., Rau I. (2016). Control of the IR-spectral shift via modification of the surface relief between the liquid crystal matrixes doped with the lanthanide nanoparticles and the solid substrate. Opt. Express.

